# Supplementary material for: Comparing DNA Isolation and Preparation Protocols for Dried Blood Spots in the Context of Genomic Newborn Screening
Source: Int J Neonatal Screen. 2025 Sep 3;11(3):75. doi: 10.3390/ijns11030075 (PMC12452450; doi:10.3390/ijns11030075)
Supplement: Supplementary file 1 [file IJNS-11-00075-s001.zip › IJNS-3796442-supplementary.pdf]

Supplementary Materials

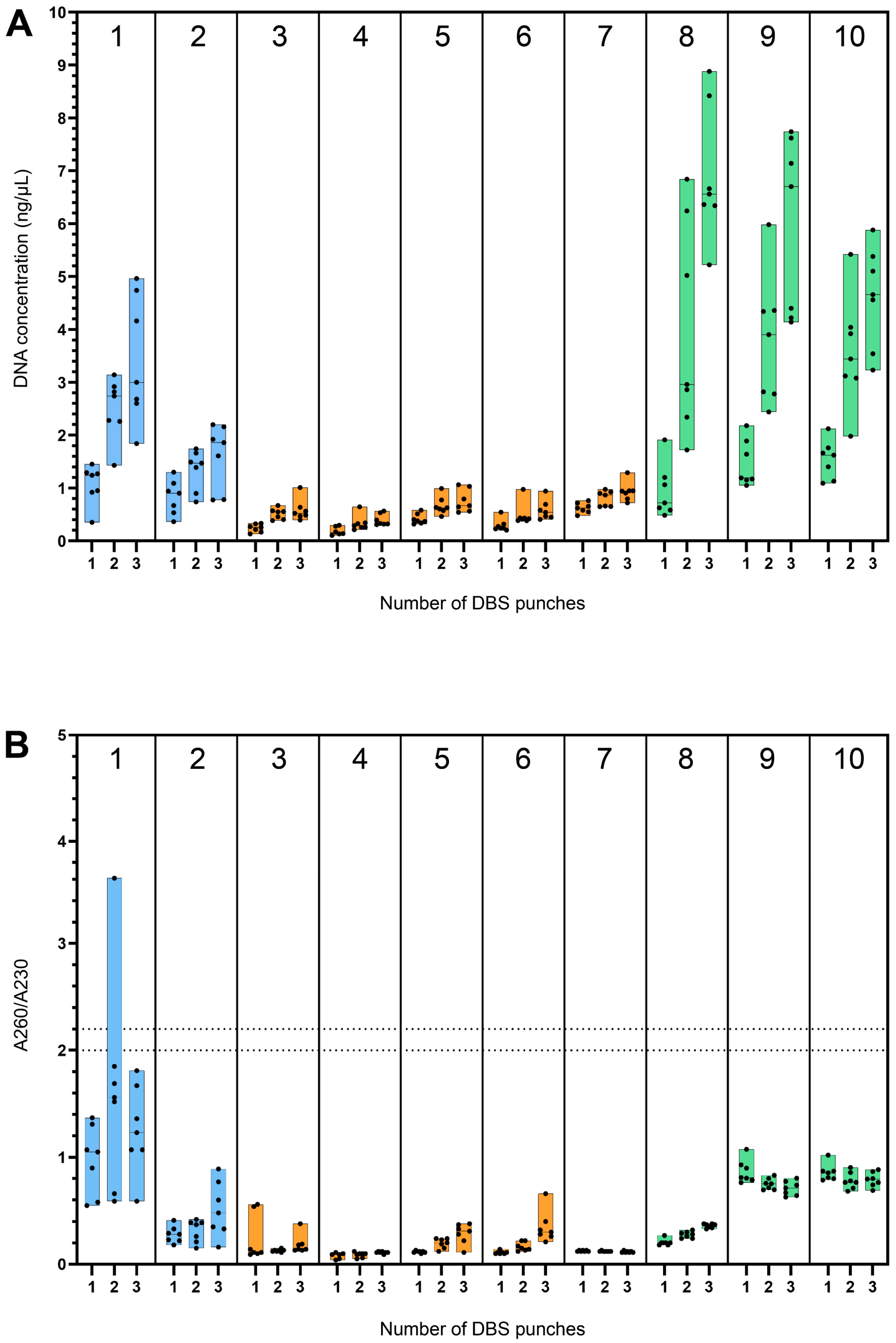

Supplemental Figure S1. DNA concentration and A260/A230 ratio

DBS = dried blood spot.

Comparison of DNA concentration (ng/µL) **(A)** and A260/A230 **(B)** ratio between ten different DNA isolation protocols using one, two, and three DBS punches (3.2 mm). The dotted lines depict the range of optimal A260/A230 ratio **(B).** The numbers above the columns correspond to the DNA isolation protocols as described in Table 1. The median and range for the measurements per protocol are depicted by the bars, with colours indicating different techniques used, including column-based (blue), lysis-based (orange), and magnetic beads-based (green).

Supplemental Table S1. Mean DNA concentration, DNA yield, and OD ratios

Protocol, punches			DNA yield in ng, mean (SD)	CV for DNA yield	95% CI for DNA yield	DNA concentration in ng/µL, mean (SD)	A260/A280, mean (SD)	A260/A230, mean (SD)
1	Adapted Sigma Mini	1	21.34 (7.38)	0.35	14.51 - 28.16	1.07 (0.37)	1.51 (0.13)	0.98 (0.32)
		2	50.26 (11.53)	0.23	39.59 - 60.92	2.51 (0.58)	1.66 (0.26)	1.64 (1)
		3	68.51 (23.88)	0.35	46.43 - 90.60	3.43 (1.19)	1.75 (0.27)	1.26 (0.41)
2	QIAamp Micro	1	16.57 (6.52)	0.39	10.54 - 22.59	0.83 (0.33)	2.62 (0.76)	0.28 (0.08)
		2	26.81 (7.59)	0.28	19.79 - 33.83	1.34 (0.38)	1.32 (0.33)	0.31 (0.11)
		3	32.30 (12.10)	0.37	21.10 - 43.49	1.62 (0.61)	1.99 (0.86)	0.51 (0.26)
3	QIAGEN ES-1	1	24.29 (7.40)	0.30	17.45 - 31.13	0.24 (0.08)	1.22 (0.19)	0.24 (0.22)
		2	51.26 (10.22)	0.20	41.81 - 60.71	0.51 (0.10)	1.06 (0.05)	0.13 (0.01)
		3	58.23 (20.33)	0.35	39.43 - 77.03	0.58 (0.20)	1.04 (0.05)	0.18 (0.09)
4	QIAGEN ES-2	1[a]	18.53 (8.12)	0.44	10.01 - 27.05	0.19 (0.08)	0.93 (0.18)	0.08 (0.03)
		2	33.17 (14.47)	0.44	19.79 - 46.56	0.33 (0.14)	0.92 (0.13)	0.09 (0.02)
		3	39.49 (10.95)	0.28	29.36 - 49.61	0.39 (0.11)	1 (0.02)	0.11 (0.01)
5	QIAGEN ES-3	1	20.66 (4.79)	0.23	16.23 - 25.08	0.41 (0.10)	1.04 (0.04)	0.12 (0.01)
		2	33.29 (8.51)	0.26	25.42 - 41.15	0.67 (0.17)	1.04 (0.03)	0.20 (0.05)
		3	37.80 (10.69)	0.28	27.91 - 47.69	0.76 (0.21)	1 (0.04)	0.29 (0.09)
6	QIAGEN ES-4	1	14.57 (5.82)	0.40	9.19 - 19.96	0.29 (0.12)	0.99 (0.05)	0.11 (0.01)
		2	24.53 (10.71)	0.44	14.63 - 34.43	0.49 (0.21)	0.97 (0.04)	0.16 (0.04)
		3	29.03 (9.24)	0.32	20.48 - 37.58	0.58 (0.18)	0.97 (0.05)	0.35 (0.15)
7	Thermo Fisher	1	24.90 (3.91)	0.16	21.28 - 28.52	0.62 (0.10)	0.99 (0.03)	0.12 (0.01)
		2	32.14 (5.76)	0.18	26.81 - 37.46	0.80 (0.14)	1.01 (0.02)	0.12 (<0.01)
		3	37.41 (7.16)	0.19	30.79 - 44.03	0.93 (0.18)	0.98 (0.03)	0.12 (0.01)
8	Maxwell	1	47.01 (25.05)	0.53	23.85 - 70.18	0.94 (0.50)	1.68 (0.14)	0.20 (0.03)
		2	199.90 (101)	0.51	106.50 - 293.20	4 (2.02)	1.66 (0.09)	0.28 (0.03)
		3	346 (63.99)	0.18	286.80 - 405.20	6.92 (1.28)	1.62 (0.06)	0.36 (0.02)
9	Chemagic Overnight	1	80.69 (24.15)	0.30	58.36 - 103	1.47 (0.44)	2.47 (0.33)	0.87 (0.11)
		2	209.20 (68.17)	0.33	146.10 - 272.20	3.80 (1.24)	1.99 (0.12)	0.75 (0.05)
		3	329.70 (91.56)	0.28	245 - 414.40	5.99 (1.67)	1.89 (0.09)	0.71 (0.07)
10	Chemagic Short	1	84.70 (20.03)	0.24	66.17 - 103.20	1.54 (0.36)	2.93 (0.32)	0.86 (0.08)
		2	196.40 (58.35)	0.30	142.50 - 250.40	3.57 (1.06)	2.14 (0.18)	0.78 (0.08)
		3	254.20 (52.66)	0.21	205.50 - 302.90	4.62 (0.96)	2.15 (0.17)	0.79 (0.07)

CI = confidence interval; CV = coefficient of variation; SD = standard deviation.

[a] For protocol 4 (‘QIAGEN ES-2’) with one DBS punch (3.2 mm) outcomes are based on six individual samples as one sample was excluded due to persistent failure despite multiple extractions.

Supplemental Table S2A-C. Post-hoc pairwise comparisons of DNA yield

A. One DBS punch (Kruskal–Wallis, *p* < 0.001)

	Adapted Sigma Mini	QIAamp Micro	QIAGEN ES-1	QIAGEN ES-2	QIAGEN ES-3	QIAGEN ES-4	Thermo Fisher	Maxwell	Chemagic Overnight	Chemagic Short
Adapted Sigma Mini	*24.80 (7.00 - 29.00)*									
QIAamp Micro	1.000	*18.04 (7.28 - 26.00)*								
QIAGEN ES-1	1.000	1.000	*25.40 (13.20 - 33.20)*							
QIAGEN ES-2	1.000	1.000	1.000	*15.40 (10.20 - 29.60)*						
QIAGEN ES-3	1.000	1.000	1.000	1.000	*19.00 (15.90 - 28.90)*					
QIAGEN ES-4	1.000	1.000	1.000	1.000	1.000	*12.00 (10.10 - 27.10)*				
Thermo Fisher	1.000	1.000	1.000	1.000	1.000	1.000	*24.80 (19.12 - 30.48)*			
Maxwell	1.000	0.112	1.000	0.507	1.000	**0.023**	1.000	*35.90 (24.20 - 95.50)*		
Chemagic Overnight	0.073	**0.002**	0.354	**0.019**	**0.041**	**0.000**	0.653	1.000	*65.45 (57.75 - 119.90)*	
Chemagic Short	0.064	**0.002**	0.314	**0.016**	**0.035**	**0.000**	0.584	1.000	1.000	*89.10 (59.95 - 116.60)*

Post-hoc pairwise comparisons after the Kruskal–Wallis test to compare DNA yield from one **(A)** DBS punch (3.2 mm) between ten different DNA isolation protocols using Dunn's test with Bonferroni correction to adjust for multiple testing. A two-sided p-value of < 0.05 was considered statistically significant. Medians and ranges for DNA yield are included in the diagonal cells crossing the same protocols.

B. Two DBS punches (Kruskal–Wallis, $p < 0.001$)

	Adapted Sigma Mini	QIAamp Micro	QIAGEN ES-1	QIAGEN ES-2	QIAGEN ES-3	QIAGEN ES-4	Thermo Fisher	Maxwell	Chemagic Overnight	Chemagic Short
Adapted Sigma Mini	*54.80 (29.60 - 62.80)*									
QIAamp Micro	1.000	*29.40 (14.72 - 34.80)*								
QIAGEN ES-1	1.000	1.000	*54.80 (38.60 - 67)*							
QIAGEN ES-2	1.000	1.000	1.000	*28.80 (21.20 - 64.40)*						
QIAGEN ES-3	1.000	1.000	1.000	1.000	*31.30 (23.10 - 49.50)*					
QIAGEN ES-4	0.461	1.000	0.272	1.000	1.000	*20.90 (19.10 - 48.70)*				
Thermo Fisher	1.000	1.000	1.000	1.000	1.000	1.000	*34.72 (25.92 - 38.96)*			
Maxwell	1.000	**0.004**	1.000	**0.021**	0.057	**0.000**	**0.046**	*148 (86 - 342)*		
Chemagic Overnight	1.000	**0.002**	1.000	**0.013**	**0.036**	**0.000**	**0.029**	1.000	*214.50 (134.20 - 328.90)*	
Chemagic Short	1.000	**0.003**	1.000	**0.017**	**0.045**	**0.000**	**0.037**	1.000	1.000	*189.20 (108.90 - 298.10)*

Post-hoc pairwise comparisons after the Kruskal–Wallis test to compare DNA yield from two **(B)** DBS punches (3.2 mm) between ten different DNA isolation protocols using Dunn's test with Bonferroni correction to adjust for multiple testing. A two-sided p-value of < 0.05 was considered statistically significant. Medians and ranges for DNA yield are included in the diagonal cells crossing the same protocols.

C. Three DBS punches (Kruskal–Wallis, $p < 0.001$)

	Adapted Sigma Mini	QIAamp Micro	QIAGEN ES-1	QIAGEN ES-2	QIAGEN ES-3	QIAGEN ES-4	Thermo Fisher	Maxwell	Chemagic Overnight	Chemagic Short
Adapted Sigma Mini	60.00 (36.80 - 99.20)									
QIAamp Micro	1.000	37.20 (15.48 - 44)								
QIAGEN ES-1	1.000	1.000	52 (39.40 - 101)							
QIAGEN ES-2	1.000	1.000	1.000	33 (30.60 - 56.40)						
QIAGEN ES-3	1.000	1.000	1.000	1.000	33.30 (27 - 53)					
QIAGEN ES-4	0.332	1.000	0.644	1.000	1.000	27 (20.30 - 47)				
Thermo Fisher	1.000	1.000	1.000	1.000	1.000	1.000	37.60 (28.80 - 51.60)			
Maxwell	1.000	**0.002**	0.936	**0.011**	**0.006**	**0.000**	**0.005**	328 (261 - 444)		
Chemagic Overnight	1.000	**0.004**	1.000	**0.027**	**0.014**	**0.000**	**0.013**	1.000	368.50 (227.70 - 425.70)	
Chemagic Short	1.000	**0.024**	1.000	0.132	0.073	**0.002**	0.070	1.000	1.000	256.30 (177.70 - 323.40)

Post-hoc pairwise comparisons after the Kruskal–Wallis test to compare DNA yield from three **(C)** DBS punches (3.2 mm) between ten different DNA isolation protocols using Dunn's test with Bonferroni correction to adjust for multiple testing. A two-sided p-value of < 0.05 was considered statistically significant. Medians and ranges for DNA yield are included in the diagonal cells crossing the same protocols.

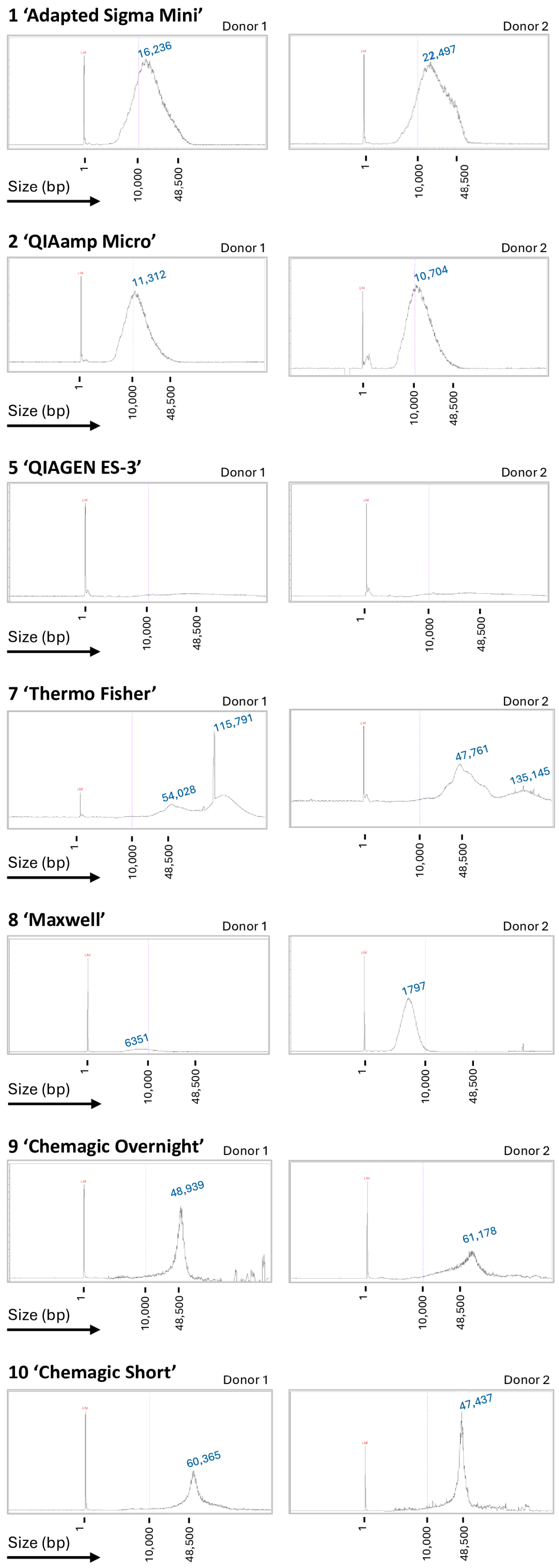

Supplemental Figure S2. Analysis of molecular weight of isolated DNA from one DBS punch

DBS = dried blood spot.

The analysis was performed with the Femto Pulse System on DNA isolated from one DBS punch (3.2 mm). For each DNA isolation protocol, results from two different donor samples are presented.

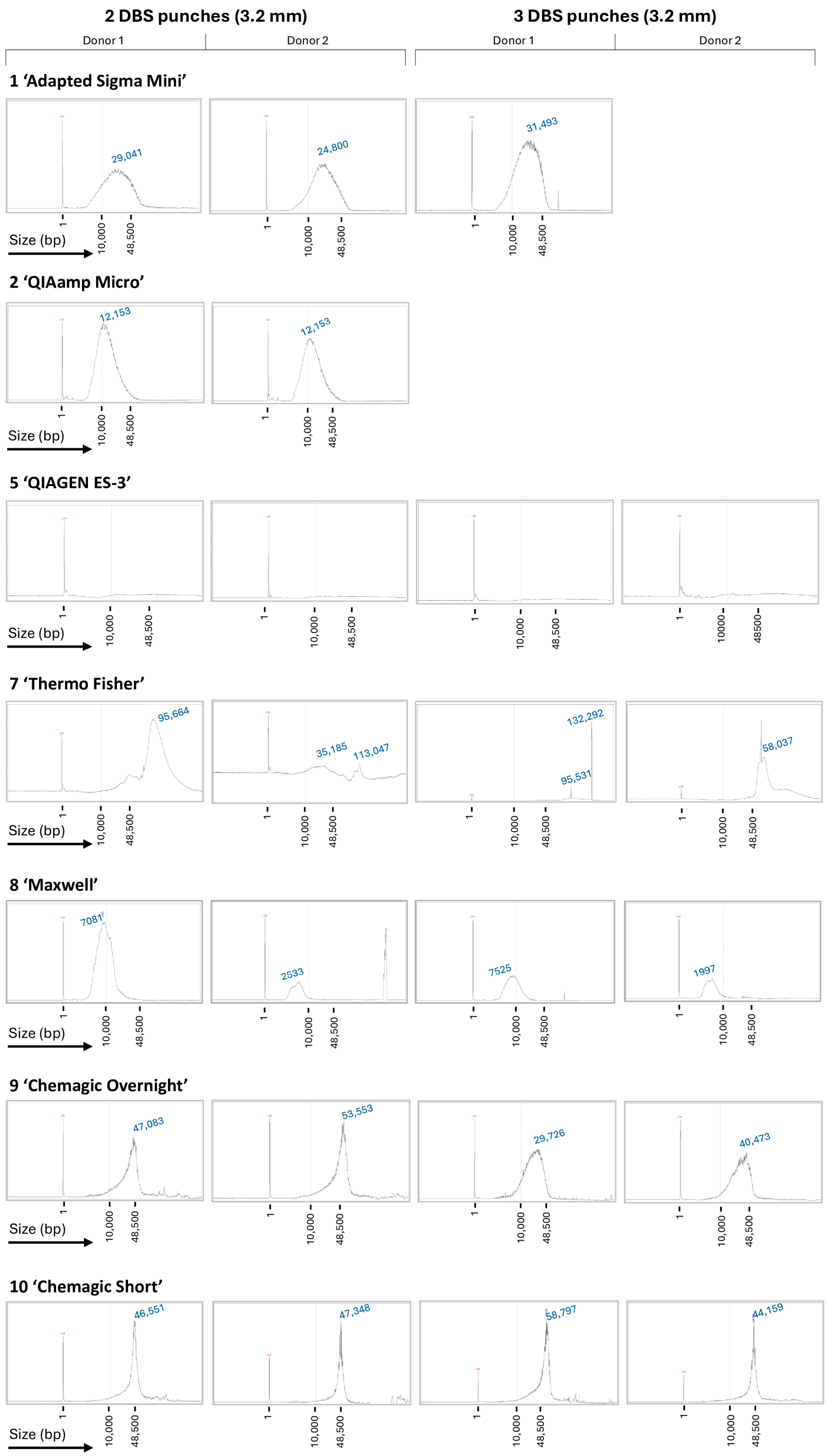

Supplemental Figure S3. Analysis of molecular weight of isolated DNA from two and three DBS punches

DBS = dried blood spot.

The analysis was performed with the Femto Pulse System on DNA isolated from two and three DBS punches (3.2 mm). For each DNA isolation protocol (rows), results from two different donor samples are presented (columns). Analysis for protocol 1 ('Adapted Sigma Mini') with three DBS punches from one donor and protocol two with three DBS punches from both donors could not be performed due to insufficient material.
